# Emotionally Unstable Personality Disorder and Severity of Suicide Attempt are related to Epigenetic Hypermethylation of Brain-Derived Neurotrophic Factor in Women

**DOI:** 10.1192/j.eurpsy.2023.765

**Published:** 2023-07-19

**Authors:** J. Jokinen, E. Jamshidi, Å. Nilsonne, A. Wilczek, A. Boström, M. Åsberg

**Affiliations:** 1 Umeå University, Umeå University, Umeå; 2 Karolinska Institutet, Stockholm, Sweden

## Abstract

**Introduction:**

Brain-derived neurotrophic factor (BDNF) has been associated with both emotionally unstable personality disorder (EUPD) and suicidal behavior. No study has yet investigated BDNF-associated epigenetic alterations in severely impaired EUPD and suicidal patients.

**Objectives:**

The main goal of the present study was to investigate whether epigenetic dysregulation in BDNF, CRP, IL-1, IL-2 and IL-6 were associated with EUPD and severity of suicidal behavior.

**Methods:**

The discovery cohort consisted of 97 women with emotionally unstable personality disorder (EUPD) with at least two serious suicide attempts (SA) and 32 healthy women. The genome-wide methylation pattern was measured by the Illumina EPIC BeadChip and analyzed by robust linear regression models to investigate mean BDNF methylation levels in a targeted analysis conditioned upon severity of suicide attempt. The validation cohort consisted of 60 female suicide attempters, stratified into low- (n=45)and high-risk groups (n=15) based on degree of intent-to-die and lethality of suicide attempt method, and occurrence of death-by-suicide at follow-up.

**Results:**

Mean BDNF methylation levels exhibited hypermethylation in relation to EUPD(p=0.0343, percentage mean group difference ~3.8%). Similarly, this locus was confirmed as hypermethylated in an independent cohort of women with severe suicidal behavior (p=0.0469). Results were independent of age and BMI.

**Conclusions:**

This study elicits emerging evidence of epigenetic dysregulation of BDNF in relation to phenotypes known to increase risk of suicide (lethality of suicide-attempt method and presence of EUPD diagnosis with history of recent SA). Further studies investigating epigenetic and genetic effects of BDNF on severe suicidal behavior and EUPD are needed to elucidate the role of epigenetic regulatory mechanisms and neurotrophic factors in relation to suicide risk.

**Disclosure of Interest:**

None Declared

